# Occupational exposure to formaldehyde and risk of lymphoma subtypes: results of a multicentre Italian case-control study

**DOI:** 10.1186/s12940-025-01232-0

**Published:** 2025-10-27

**Authors:** Pierluigi Cocco, Federico Meloni, Carlotta Trobbiani, Lucia Miligi, Daniela Ferrante, Marina Padoan, Giovanni Maria Ferri, Angela Gambelunghe, Giacomo Muzi, Corrado Magnani, Angelo Palmas, Giovanna Piras, Sara Piro, Mariagrazia Zucca, Maria Grazia Ennas, Roberta Zanotti, Aldo Scarpa, Sara De Matteis

**Affiliations:** 1 Centre for Occupational and Environmental Health, Division of Population Health, Health Services Research & Primary Care, University of Manchester, Manchester, M13 9PL United Kingdom; 2 Department of Medical Sciences and Public Health, University of Cagliari, Monserrato (Cagliari), Italy; 3 Department of Health Sciences, State University of Milan, Milano , Italy; 4 Environmental and Occupational Epidemiology Branch - Cancer Risk Factors and Lifestyle Epidemiology Unit, Institute for Cancer Research, Prevention and Clinical Network (ISPRO), Florence, Italy; 5 Department of Translational Medicine, University of Eastern Piedmont, Novara, Italy; 6 Interdisciplinary Department of Medicine (DIM), Unit of Occupational Medicine, University of Bari, Bari, Italy; 7 Department of Medical and Surgical Sciences, University of Bologna, Bologna, Italy; 8 Section of Occupational Medicine, Department of Medicine, University of Perugia, Perugia , Italy; 9 Unit of Haematology, San Francesco Hospital, Nuoro, Italy; 10 Department of Medicine, Haematology Unit, Department of Diagnostics and Public Health-Section of Pathology, Verona University Hospital, Verona , Italy; 11 Local Health Unit 7, Carbonia, Italy; 12 Department of Biomedical Sciences, University of Cagliari, Cagliari, Italy; 13 Department of Public Health and Pediatrics, University of Turin, Turin, Italy; 14 Centre for Occupational and Environmental Health, Division of Population Health, Health Services Research & Primary Care, University of Manchester, Manchester, M13 9PL UK

**Keywords:** Formaldehyde, Lymphoma, Multiple myeloma, Case-control study, Occupational exposure, Occupational cancer

## Abstract

**Background:**

The International Agency for Research on Cancer classifies formaldehyde as a human carcinogen, with sufficient evidence for nasopharyngeal cancer and leukaemia. However, the association with lymphoma subtypes has been less thoroughly investigated. We explored this link in an Italian multicentre case-control study.

**Methods:**

A total of 867 incident lymphoma cases, histologically confirmed using the WHO classification, and 774 controls participated in the study. Occupational experts classified the probability, frequency, and intensity of exposure to formaldehyde for each study subject based on detailed questionnaire data and literature information. We used unconditional regression analysis to model the risk of lymphoma and its main subgroups and subtypes associated with different formaldehyde exposure metrics, adjusting for age, gender, education, and study centre.

**Results:**

Ever exposure to formaldehyde was not associated with risk of all lymphomas combined, the non-Hodgkin lymphoma (NHL) and B-cell lymphoma (BCL) subgroups, or the most prevalent BCL subtypes, but multiple myeloma (MM) (OR = 2.0, 95% CI 1.19–3.31). MM risk also showed consistent upward trends with all the exposure metrics (*p* for trend ranging 0.005–0.034). Hodgkin’s lymphoma risk was also elevated in the top categories of intensity, duration, and cumulative exposure, but no significant increasing trends were observed.

**Conclusions:**

Our findings suggest an increased risk of multiple myeloma associated with occupational exposure to formaldehyde. A stronger link was observed for daily exposure lasting 20 years or more. The risk of Hodgkin’s lymphoma was also elevated at high exposure intensities.

**Supplementary Information:**

The online version contains supplementary material available at 10.1186/s12940-025-01232-0.

## Introduction

The International Agency for Research on Cancer (IARC) classifies formaldehyde as a human carcinogen (Group 1) with sufficient evidence of an association with risk of rhino-pharyngeal cancer and leukaemia, and a note on positive association also with cancer of the nasal sinuses [1]. The IARC Monograph 100 F Working Group observed that several other cancer sites had shown statistically significant increases in risk, including Hodgkin’s lymphoma (HL) and multiple myeloma (MM); however, studies were too few and the results were inconsistent [1]. Three studies among those published at the time of the IARC Monograph 100 F Working Group meeting explored the risk of lymphohematopoietic cancer associated with occupational exposure to formaldehyde. One was a large cohort study of workers engaged in the production and use of formaldehyde before 1966, which showed an increase in mortality from all lymphohematopoietic cancer (*p* for trend = 0.02) and HL (*p* for trend = 0.01) with quantitative estimates of formaldehyde exposure. Risk associated with peak exposures ≥ 4 ppm was also elevated for MM, although there was no significant upward trend [2]. The second study was a population-based case-control investigation of MM among Danish women, whose occupational exposure was assessed by industrial hygienists based on occupational titles. Women with probable exposure to formaldehyde had a non-significant 1.6-fold increase in risk [3]. In the subsequent years, a new cohort study investigated the mortality experience among employees of an Italian laminated plastic factory exposed to formaldehyde for at least 180 days, followed up from 1947 to 2011 [4]. All deaths and cancer deaths were significantly less than expected; nine cases of cancer of the oral cavity and pharynx occurred vs. 6.1 expected, of which three were cases of nasopharyngeal cancer. Deaths from lymphohematopoietic cancer were also below expectation, with four cases of lymphoma (Hodgkin and non-Hodgkin combined), five cases of leukaemia (all forms combined), and none of MM. All lymphoma deaths occurred among workers employed 10 years or more (SMR = 147, *p* value not reported), and leukaemia deaths were concentrated among those first exposed after age 35 years and decreased with years since last exposure [4]. In a new analysis of the U.S. National Cancer Institute (NCI) cohort data on lymphohematopoietic malignancies [5], acute myeloid leukaemia was unrelated to cumulative exposure to formaldehyde; chronic myeloid leukaemia trends suggested an association but based on few deaths; and HL risk increased with cumulative and peak exposure up to 3.5-fold (*p* for trend = 0.003). There was no association with other lymphohematopoietic malignancies [5]. Finally, a meta-analysis of 12 studies did not find an association with the risk of non-Hodgkin lymphoma (NHL) [6], whilst a systematic review of 21 studies supported a weak association with lung cancer, nasopharyngeal cancer, leukaemia, and NHL [7].

Lymphomas are a heterogeneous group of malignancies originating from immune system cells at various stages of their development. Each lymphoma subtype results from a specific set of complex and multifactorial aetiological combinations, including gene polymorphisms, immune system disorders, infectious agents, and workplace and environmental factors [8]. Such complexity makes it difficult to interpret results based on generic definitions of the disease entity.

The aim of this analysis of data from an Italian multicentre case-control study was to explore the association between occupational exposure to formaldehyde and the risk of all lymphoma combined, the NHL and B-cell lymphoma (BCL) subgroups, and the most represented BCL subtypes, including diffuse large B-cell lymphoma (DLBCL), follicular lymphoma (FL), chronic lymphocytic leukaemia (CLL), MM, and HL.

## Methods

The Italian multicentre case-control study on the aetiology of lymphoma took place in six centres, including the provinces of Cagliari and Nuoro in Sardinia and Bari and Taranto in Apulia, and the cities of Perugia, Florence, Novara, and Verona, between 2011 and 2017. The study population consisted of incident lymphoma cases and age- and sex-frequency-matched hospital controls in the centres of Apulia, Perugia, Novara, and Verona. Controls were selected among patients admitted for diseases unrelated to known lymphoma risk factors. Exclusion and inclusion criteria for hospital controls are listed in Additional file 1. In the centres of Cagliari, Nuoro, and Florence, population controls were randomly selected among residents in the study areas, also frequency-matched to cases by sex and 5-year age group. All cases were histologically confirmed diagnoses of lymphoma (any subtype) first admitted to the participating oncohaematology units in each centre during the study period, classified with the 2008 update of the WHO classification of lymphoma [9]. This classification includes plasma cell myeloma (ICDO3 code 9732/3, WHO 2008 code 9733/3, corresponding to the multiple myeloma/plasma cell leukaemia definition adopted in the 2001 WHO classification [10]) and chronic lymphocytic leukaemia (ICDO3 code 9823/3, WHO 2008 code 9670/3) among the mature B-cell lymphomas. Overall, 867 lymphoma cases, including 105 DLBCL, 81 CLL, 87 FL, 95 MM, and 181 HL, and 774 controls (340 hospital controls and 434 population controls) were included in the analysis.

Due to a higher refusal rate among the controls (38.4% vs. 7.4% among the cases), the case-control matching lost its effectiveness and, therefore, we proceeded using an unmatched analysis.

Trained interviewers administered the questionnaire to all study participants at the hospital or their residences. Besides demographic, lifestyle, and health history information, the questionnaire included a complete work history. Study subjects had to list all jobs lasting at least one year, describing the type of industry, its activity, the tasks they were entitled to, and the tools they used, along with a subjective judgment about the occurrence of a list of exposures of interest. Specific job modules were applied when jobs were reported that were suspected of conveying exposure to risk factors of a priori interest, based on the existing literature, to gather more detailed information. Expert occupational physicians and industrial hygienists evaluated formaldehyde exposure based on the work history of study subjects, the information reported in the job modules, the existing literature [11–14], and personal experience. Exposure was classified using the following metrics:

1. probability, which indicates the degree of industrial hygienists’ confidence that the subject had indeed been exposed to formaldehyde, based on two criteria: (1) a summary evaluation of the probability of exposure (1 = possible but not probable; 2 = probable; and 3 = certain); and (2) proportion of exposed in that specific job (1 = ≤ 40%; 2 = 40–90%; 3 = ≥ 90%);
2. intensity of exposure, based on the exposure circumstances and the use of personal protective equipment.
3. frequency of exposure, in semi-quantitative terms (daily, 1–4 days/week, 1–3 days/month).

Cumulative exposure was expressed as a score by applying the formula below:

$${\mathrm C}_{\mathrm i}=\sum{({\mathrm y}_{\mathrm j}\times{\mathrm f}_{\mathrm j}/3)}^{{\mathrm x}_{\mathrm j}}$$

where:

C_i_= cumulative exposure score.

i = study subject.

j = *jth* job entry in the work history of the *ith* study subject.

y = duration of exposure (in years).

x = exposure intensity level.

f = exposure frequency level.

Table 1 shows the occupations associated with the various degrees of probability and intensity of exposure to formaldehyde. As the work organisation and, consequently, the time on specific tasks in the same job can vary by workplace, exposure frequency is not included in Table 1.

**Table 1:** List of occupations by probability and intensity of formaldehyde exposure

Intensity*	Probability		
	Possible	Probable	Certain
Low (up to 0.5 ppm)	Nurse in the military, biology teacher, furniture salesman, bricklayer, construction labourer, textile industry supervisor, cheese-making supervisor, tire rebuilder, electrical circuits and wiring manufacturer.	Neonatologist, orthopaedic, waterproof coats’ sewer, fur clothes sewer.	Anaesthetist, foundryman, polyurethane foam insulator.
Medium (0.5–1.5 ppm)	Hospital physician, hospital nurse, food shop salesman, bakery cleaner, street food salesman, dog shelter attendant, housekeeper, cook and cook helper, janitor (including school), cleaning personnel, butcher, plastic moulder.	Clinical laboratory technician, nurse, dentist, dentist’s assistant, tile setter. cabinet maker.	orthodontic technician, umbrella manufacturer, leather shoe finisher, antibiotic manufacturer, parquetry worker.
High (> 1.5 ppm)	Photographer, photography teacher, butcher shop, detergent manufacturer, chair upholsterer.	Microbiology laboratory technician, assistant nurses in a hospital, hairdresser, furniture and plywood maker, weaving finisher, cheese maker, shoemaker.	Pathology laboratory technician, operating room nurses, assistant nurse in autopsy room, beautician, manicurist, chemical blender in textile industry, leather tanning, leather purse manufacturer, leather curing and dying.

### Statistical methods

We used unconditional logistic regression models to predict the risk of lymphoma (all combined), NHL (including T and B-cell lymphomas, but chronic lymphocytic leukaemia and multiple myeloma), BCL and its most represented subtypes, and HL by probability, intensity level, frequency, duration and cumulative exposure to formaldehyde. Each model included age, sex, study centre, and education (primary school, middle-high school, university degree, or technical education as the adjusting covariates. Ever-exposure to solvents, farm work, marital status, body mass index, smoking, and alcohol were also considered. However, in a preliminary analysis, none of these covariates showed an association with the risk of lymphoma or its subgroups and subtypes. Including or excluding each of them from the regression model did not change the risk estimates or reduce the residual variance, which would exclude their role as confounders in the association with formaldehyde. In all analyses, the reference category included subjects who had never been exposed to formaldehyde.

The risk associated with various degrees of probability, intensity (average or top level over the work history), frequency (average or top level over the work history), and duration (quartiles or pre-determined categories of exposure: ≤ 10, 11–20, 21–35, or ≥ 36 years) was calculated as the Odds Ratio (OR). We calculated two-tailed 95% confidence intervals (95% CI) using Wald’s formula (e^*β*^ *±* (*z*_α/2_ * *se*_*β*_)).

The Ethics Committee of the Cagliari University Hospital approved the study protocol on 26 May 2009 (Protocol No. 269/09/CE). Local Ethics Committees approved the study in each of the participating study centres. All participants provided written consent in accordance with the Helsinki declaration.

## Results

Table 2 shows selected characteristics of the study population by case-control status. Men were slightly more prevalent among the cases, and women among the controls. The participants’ mean age was 55.0 years (standard deviation [*sd*] 15.4), similar by case-control status, and slightly elevated among men (56.6 years, *sd* 14.6) compared to women (53.0 years, *sd* 16.2) (*p* < 0.001). Cases were less educated than controls. Ever exposure to formaldehyde was more prevalent among the cases (20.9%) than the controls (17.3%) (*p* = 0.067).

[Table 2]

**Table 2:** Selected characteristics of the study population

Variable	Cases (No. = 867) *N* %	Controls (No. =774) *N* %	Total (No. = 1641) *N* %
Sex			
Men	500 (57.6)	428 (55.3)	928 (56.6)
Women	367 (42.4)	346 (44.7)	713 (43.4)
M/F ratio	1.4	-	-
Study area			
Cagliari	131 (15.1)	178 (23.0)	309 (18.8)
Nuoro	75 (8.7)	49 (6.3)	124 (7.6)
Verona	31 (3.6)	68 (8.8)	99 (6.0)
Firenze	228 (26.3)	188 (24.3)	416 (25.4)
Bari	132 (15.2)	70 (9.0)	202 (12.3)
Taranto	78 (9.0)	38 (4.9)	116 (7.1)
Novara	102 (11.8)	85 (11.0)	187 (11.4)
Perugia	90 (10.4)	98 (12.7)	188 (11.4)
Education			
None	15 (1.7)	11 (1.4)	26 (1.6)
Elementary	167 (19.3)	107 (13.8)	274 (16.7)
Middle school	262 (30.2)	231 (29.8)	493 (30.0)
High school	265 (30.6)	284 (36.7)	549 (33.4)
University	123 (14.2)	122 (15.8)	245 (14.9)
Other	35 (4.0)	19 (2.5)	54 (3.4)
Marital status			
Single	155 (17.9)	153 (19.8)	308 (18.8)
Married	513 (59.2)	433 (55.9)	946 (57.6)
Divorced	46 (5.3)	43 (5.6)	89 (5.4)
Widow	47 (5.4)	36 (4.7)	83 (5.1)
Not reported	106 (12.2)	109 (14.1)	215 (13.1)
Body mass index			
≤ 25	441 (50.9)	349 (45.1)	790 (48.1)
25.1–30	304 (35.1)	304 (39.3)	608 (37.1)
≥ 30.1	122 (14.1)	121 (15.6)	243 (14.8)
Smoking			
Never	410 (47.3)	368 (47.5)	778 (47.4)
Current smokers	175 (20.2)	159 (20.5)	334 (20.4)
Ex-smokers	272 (31.4)	240 (31.0)	512 (31.2)
Not reported	10 (1.1)	7 (0.9)	17 (1.0)
Alcohol intake			
Abstinent	424 (48.9)	382 (49.4)	806 (49.1)
Daily	237 (27.3)	214 (27.6)	451 (27.5)
Occasional	55 (6.3)	52 (6.7)	107 (6.5)
Ex-drinker	43 (5.0)	34 (4.4)	77 (4.7)
Not reported	108 (12.5)	92 (11.9)	200 (12.2)
Occupational exposure to formaldehyde			
Never	686 (79.1)	640 (82.7)	1325 (80.7)
Ever	181 (20.9)	134 (17.3)	316 (19.3)

Table 3 shows the OR for all lymphoma combined, NHL, BCL, BCL subtypes, and HL associated with ever exposure, as well as the probability of formaldehyde exposure. A significant association was only observed for MM (OR = 2.0, 95% CI 1.19–3.30), which also showed a significant upward trend with the probability of exposure (*p* = 0.017).

[Table 3]

**Table 3:** Odds ratio for lymphoma and its most represented subtypes by probability of exposure to formaldehyde. Covariates in the logistic regression model include age, sex, study centre, and education

Case Subset	Unexposed	Ever exposed	*Probability*			
			*low*	*medium*	*high*	*p* test for trend
	*Ca/co OR*	* Ca/co OR* *95% CI*	* Ca/co OR* *95% CI*	* Ca/co OR* *95% CI*	* Ca/co OR* *95% CI*	
All lymphomas	686/640 1.0	181/134 1.2 0.90-1.49	104/84 1.1 0.77-1.44	52/36 1.2 0.79-1.92	25/14 1.5 0.76-2.90	0.098
Non-Hodgkin’s lymphoma	391/640 1.0	90/134 1.1 0.78-1.44	53/84 1.0 0.69-1.48	23/36 1.0 0.56-1.68	13/14 1.4 0.65-3.10	0.206
B-cell lymphoma	378/640 1.0	90/134 1.0 0.75-1.40	46/84 0.9 0.57-1.28	28/36 1.1 0.67-1.92	15/14 1.5 0.71-3.31	0.253
Diffuse Large B-cell lymphoma	84/640 1.0	21/134 1.0 0.61-1.77	13/84 1.0 0.54-1.99	3/36 0.6 0.17-1.87	5/14 2.2 0.74-6.25	0.215
Follicular lymphoma	75/640 1.0	12/134 0.8 0.42-1.50	6/84 0.6 0.24-1.39	4/36 0.9 0.31-2.62	2/14 1.1 0.23-4.83	0.399
Chronic Lymphocytic Leukaemia	68/640 1.0	13/134 0.8 0.44-1.61	6/84 0.6 0.25-1.49	6/36 1.5 0.60-3.76	1/14 0.6 0.07-4.59	0.382
Multiple Myeloma	65/640 1.0	30/134 2.0 1.19-3.30	18/84 1.8 0.96-3.33	8/36 2.3 1.00-5.41	4/14 2.3 0.66-7.77	0.017
Hodgkin’s lymphoma	140/640 1.0	41/134 1.4 0.87-2.15	23/84 1.1 0.65-1.99	12/36 1.8 0.84-3.79	6/14 1.9 0.63-5.65	0.136

*Ca *cases,* Co *controls

The analysis by different exposure metrics consistently showed elevated ORs for MM. Daily exposure was associated with a 6-fold increase in MM risk (95% CI 1.84–19.3), based on 6 cases and 11 controls), again with a significant upward trend (*p* for trend = 0.005).

[Table 4]

**Table 4:** Odds ratio for lymphoma and its most represented subtypes by average frequency of exposure to formaldehyde. Covariates in the logistic regression model include age, sex, study centre, and education

Case subset	Unexposed	Average frequency			
		1-3 times/month	1-5 times/week	daily	p test for trend
	Ca/co OR	Ca/co OR 95% CI	Ca/co OR 95% CI	Ca/co OR 95% CI	
All lymphomas	686/640 1.0	106/88 1.0 0.74-1.38	54/35 1.3 0.84-2.05	21/11 1.6 0.74-3.31	0.056
Non-Hodgkin’s lymphoma	391/640 1.0	55/88 1.0 0.68-1.42	28/35 1.3 0.74-2.13	6/11 0.9 0.32-2.46	0.195
B-cell lymphoma	378/640 1.0	60/88 0.9 0.72-1.51	16/35 0.7 0.37-1.30	13/11 1.8 0.78-4.29	0.324
Diffuse Large B-cell lymphoma	84/640 1.0	17/88 1.3 0.71-2.29	2/35 0.4 0.09-1.65	2/11 1.2 0.25-5.45	0.355
Follicular lymphoma	75/640 1.0	9/88 0.8 0.40-1.78	1/35 0.2 0.03-1.59	2/11 1.5 0.32-6..99	0.380
Chronic Lymphocytic Leukaemia	68/640 1.0	8/88 0.8 0.35-1.70	4/35 1.0 0.34-3.00	1/11 0.9 0.11-7.84	0.335
Multiple Myeloma	65/640 1.0	18/88 1.8 0.99-3.37	6/35 1.4 0.54-3.65	6/11 6.0 1.84-19.3	0.005
Hodgkin’s lymphoma	140/640 1.0	21/88 1.1 0.62-1.90	15/35 2.0 0.95-4.25	5/11 1.0 0.27-3.56	0.174

*Ca* cases, *Co* controls

Results were similar when using the top frequency of exposure to formaldehyde over the work history as the exposure variable (*p* for trend = 0.025) (Additional file 2)

Regarding exposure intensity, the OR for MM was significantly elevated at low (OR = 2.0, 95% CI 1.05–3.73) and medium-average intensity (OR = 2.9, 95% CI 1.26–6.85); there were no cases and 12 controls with high-level exposure (Additional file 3). The OR for Hodgkin’s lymphoma was elevated in the medium and high intensity categories (OR = 2.0, 95% CI 0.99–4.99 and OR = 2.1, 95% CI 0.62–7.00, respectively), with a non significant trend (*p* for trend = 0.055). After combining the medium and high average exposure intensity categories, the OR for MM was 2-fold (95% CI 0.91–4.30), and the upward trend was significant (*p* for trend = 0.034) (Table 5). HL showed an association for medium and high average intensity categories combined (OR = 2.0, 95% CI 1.08–3.80), but there was no evidence of an increasing trend.

[Table 5]

**Table 5:** Odds ratio for lymphoma and its most represented subtypes by average intensity of exposure to formaldehyde. Covariates in the logistic regression model include age, sex, study centre, and education. Medium and high exposure intensity categories are combined

Case Subset	Unexposed	*Average intensity*		
		*Low*	*Medium-high*	*p* test for trend
	*Ca/co OR*	* Ca/co OR* *95% CI*	* Ca/co OR* *95% CI*	
All lymphomas	686/640 1.0	103/86 1.0 0.72-1.34	78/48 1.4 0.96-2.07	0.067
Non-Hodgkin’s lymphoma	391/640 1.0	56/86 1.0 0.68-1.42	33/48 1.1 0.69-1.78	0.215
B-cell lymphoma	378/640 1.0	61/86 1.1 0.74-1.55	28/48 0.9 0.55-1.50	0.393
Diffuse Large B-cell lymphoma	84/640 1.0	17/86 1.3 0.71-2.32	4/48 0.6 0.20-1.68	0.337
Follicular lymphoma	75/640 1.0	10/86 1.0 0.47-1.97	2/48 0.3 0.08-1.36	0.229
Chronic Lymphocytic Leukaemia	68/640 1.0	6/86 0.6 0.23-1.38	7/48 1.4 0.60-3.34	0.396
Multiple Myeloma	65/640 1.0	20/86 2.0 1.09-3.61	10/48 2.0 0.91-4.31	0.034
Hodgkin’s lymphoma	140/640 1.0	19/86 0.9 0.49-1.62	22/48 2.0 1.08-3.80	0.088

* Ca *case*s, Co *controls

When exploring the top intensity of exposure, the OR for MM was significantly elevated at low (OR = 2.0, 95% CI 1.05–3.73) and medium average intensity (OR = 2.9, 95% CI 1.26–6.85). The high intensity category included only one MM case and 30 controls, but the trend in MM risk was still significant (Additional file 4). After combining medium and high top exposure intensity, the OR for MM was significantly elevated (OR = 2.2, 95% CI 1.23–4.12), and the upward trend was confirmed (*p* = 0.010) (Additional file 5). HL showed a non-significant association and, again, no trend was observed.

MM and HL ORs also increased by years of exposure to formaldehyde, whether categorised by pre-defined cut-offs (< 10, 11–20, 21–35, ≥ 36 years) or quartiles, and were both significantly elevated for exposure lasting 36 years or more (OR = 2.6, 95% CI 1.14–2.86, and OR = 3.6, 95% CI 1.23–10.5, respectively). Again, an upward trend was only observed for MM (*p* for trend = 0.005) (Table 6, Additional file 6). [Table 6]. 

**Table 6:** Odds ratio for lymphoma and its most represented subtypes by years of exposure to formaldehyde. Covariates in the logistic regression model include age, sex, study centre, and education

Case Subset	Unexposed	*Years of exposure*				
		1-10 years	11-20 years	21-35 years	≥ 36 years	*p* test for trend
	Ca/co OR	Ca/co OR 95% CI	Ca/co OR 95% CI	Ca/co OR 95% CI	Ca/co OR 95% CI	
All lymphomas	686/640 1.0	78/58 1.2 0.81-1.68	31/24 1.1 0.61-1.84	36/30 1.1 0.64-1.74	36/22 1.3 0.77-2.31	0.075
Non-Hodgkin’s lymphoma	391/640 1.0	35/58 1.1 0.69-1.71	18/24 1.2 0.60-2.17	18/30 0.9 0.50-1.59	18/22 1.0 0.54-1.99	0.183
B-cell lymphoma	378/640 1.0	36/58 1.2 0.76-1.91	15/24 0.9 0.44-1.74	17/30 0.8 0.42-1.46	21/22 1.1 0.57-1.99	0.367
Diffuse Large B-cell lymphoma	84/640 1.0	13/58 1.4 0.69-2.90	6/24 1.5 0.58-3.86	2/30 0.4 0.10-2.94	2/22 0.5 0.11-2.24	0.392
Follicular lymphoma	75/640 1.0	6/58 1.0 0.40-2.41	1/24 0.3 0.04-2.37	3/30 0.8 0.23-2.59	2/22 0.6 0.13-2.51	0.348
Chronic Lymphocytic Leukaemia	68/640 1.0	5/58 1.0 0.38-2.71	3/24 1.0 0.29-3.67	3/30 0.8 0.23-2.74	2/22 0.5 0.12-2.27	0.236
Multiple Myeloma	65/640 1.0	9/58 2.0 0.87-4.42	3/24 1.1 0.29-3.88	8/30 2.0 0.86-4.83	10/22 2.6 1.14-2.86	0.005
Hodgkin’s lymphoma	140/640 1.0	27/58 1.4 0.79-2.40	5/24 0.9 0.31-2.45	4/30 1.2 0.38-3.47	5/22 3.6 1.23-10.4	0.090

*Ca *cases,* Co *controls

The Odds ratio for MM did not increase linearly by cumulative exposure; the highest was observed in the medium-low category (OR = 3.5, 95% CI 1.69–7.27). Then, it decreased in the medium-high, and increased again up to 1.6-fold in the high cumulative exposure category (Table 7). The non-linear increase in the OR was substantially confirmed in the analysis by quartiles of cumulative exposure (Supplementary Table 7). Nevertheless, the upward trend was again significant, whether using pre-defined cutoffs or quartiles (*p* = 0.020 and *p* = 0.013, respectively) (Table 7 and Supplementary Table 7). The OR for HL was also elevated in the top category of cumulative exposure (OR = 3.3, 95% CI 1.29–8.61) with no evidence of a trend. The analysis by quartiles confirmed the results. Once again, neither lymphoma (all subtypes combined) nor lymphoma subgroups or other subtypes showed an association.

[Table 7]

**Table 7:** Odds ratio for lymphoma and its most prevalent subtypes by pre-defined categories of cumulative formaldehyde exposure. Covariates in the logistic regression model include age, sex, study centre, and education

Case Subset	Unexposed	Cumulative exposure score				
		low	–medium-low	–medium-high	high	*p*test for trend
	*Ca/co OR*	* Ca/co OR* *95% CI*	* Ca/co OR* *95% CI*	* Ca/co OR* *95% CI*	* Ca/co OR* *95% CI*	
All lymphomas	686/640 1.0	54/42 1.1 0.70-1.64	50/36 1.2 0.77-1.89	38/32 1.0 0.62-1.66	29/24 1.4 0.81-2.32	0.059
Non-Hodgkin’s lymphoma	391/640 1.0	29/42 1.2 0.71-1.96	20/36 0.9 0.49-1.54	23/32 1.2 0.66-2.03	17/24 1.0 0.50-1.81	0.1197
B-cell lymphoma	378/640 1.0	26/42 1.1 0.62-1.81	29/36 1.2 0.74-2.10	14/32 0.7 0.34-1.30	20/24 1.0 0.55-1.90	0.367
Diffuse Large B-cell lymphoma	84/640 1.0	9/42 1.4 0.65-3.18	6/36 1.1 0.45-2.76	3/32 0.6 0.19-2.16	3/24 0.8 0.22-2.69	0.333
Follicular lymphoma	75/640 1.0	6/42 1.3 0.52-3.29	4/36 0.9 0.30-2.59	0/32 - -	2/24 0.5 0.12-2.38	0.212
Chronic Lymphocytic Leukaemia	68/640 1.0	5/42 1.1 0.41-3.08	2/36 0.5 0.11-2.06	1/32 0.3 0.04-2.22	5/24 1.5 0.53-4.05	0.335
Multiple Myeloma	65/640 1.0	3/42 0.8 0.21-2.68	14/36 3.4 1.67-7.06	7/32 1.9 0.77-4.87	6/24 1.6 0.62-4.31	0.006
Hodgkin’s lymphoma	140/640 1.0	15/42 1.0 0.51-2.03	14/36 1.9 0.92-3.92	5/32 0.7 0.26-2.00	7/24 3.3 1.29-8.61	0.138

When stratifying the analysis by the type of controls, the OR for MM was elevated with population controls (OR = 3.4, 95%CI 1.70–6.61) but not hospital controls (OR = 1.3, 95%CI 0.86–2.89). After conducting a sensitivity analysis by excluding hospital workers, a group frequently experiencing formaldehyde exposure, the OR for MM was still 2.1-fold (95%CI 1.22–3.50) for those ever exposed, 2.5-fold for those most likely exposed (95%CI 0.69–8.78), 5.6-fold (95%CI 1.56–20.1) for the highest exposure frequency category, and 3.2-fold (95%CI 1.50–6.70) for the upper category of exposure duration (data not shown).

We explored cohort effects by stratifying the analysis by decade of starting exposure whether up to 1960, between 1961 and 1970, 1971–1980, 1981–1990, 1991–2000, or from 2001 onwards. This analysis showed that the MM OR was not elevated for exposure to formaldehyde that initiated before 1960 (OR = 1.0, 95%CI 0.35–3.01) or after 1990 (1991–2000: OR = 0.7, 95%CI 0.08–5.21; no cases were exposed after 2000), and it was 3.4-fold (95%CI 1.57–7.34) for exposure initiated in 1961-70, 3.0-fold (95% CI 1.22–7.28) in 1971-80, and 1.8-fold (95%CI 0.57–5.93) in 1981-90 (data not shown).

As it concerns HL, the OR was also elevated for exposure starting in 1961–1970 (OR = 3.2, 95%CI 1.08–9.28), and in 1971–1980 (OR = 2.0, 95%CI 0.64–6.30), but not before or after those time windows (data not shown).

We explored latency effects by stratifying the analysis by years since formaldehyde exposure ceased. MM OR ranged 1.7-2.0-fold for exposure ending between less than 5 and more than 20 years before diagnosis. The OR for HL for formaldehyde exposure ceasing 10–14 years before diagnosis was also elevated (OR = 4.2, 95% CI 1.35–13.2), but not for more distant or recent exposures. (Additional file 8).

## Discussion

Our results suggest that daily exposure to formaldehyde prolonged for 21 or more years conveys an increased risk of developing multiple myeloma. The intensity of exposure and cumulative exposure also showed an association, but not a monotonic upward trend. The Odds Ratios were relatively consistent across the exposure metrics. Assuming a true underlying association, this could have resulted from an imperfect categorisation of formaldehyde exposure, or suggests that there was difficulty in ensuring independence across the exposure metrics. In the absence of a true association, unidentified confounders well correlated with occupational exposure to formaldehyde might have generated our positive findings. Hodgkin’s lymphoma also showed some signal of an excess risk, but there was a less consistent pattern of association. Overall, the ORs for lymphoma (all combined), NHL and BCL subgroups, and the DLBCL, FL, and CLL subtypes was unrelated to formaldehyde exposure.

Our results are consistent with a proportional mortality study among U.S. embalmers [15] and a Danish case-control study of multiple myeloma among women [3]. In the NCI formaldehyde cohort, the risks of HL and MM increased with peak exposure above 2 and 4 ppm, respectively. However, the risk was not elevated in the total exposed subcohort, and there was no evidence of an upward trend with the number of such peak exposures or duration of exposure [2]. A re-analysis of the same cohort using Cox’ proportional hazard models confirmed the excess risk of HL, but not MM, associated with peak exposure to formaldehyde [5]. However, such analysis used the unexposed sub-cohort as the reference, whose mortality from MM was almost threefold the expected based on national mortality statistics in the U.S.A [2, 5]. On the other side, the observed deaths from MM were less than expected in an Italian cohort of laminated plastic manufacturers [4].

Also in agreement with our findings, a meta-analysis on the association with the NHL group did not observe an association with exposure to formaldehyde, independent of exposure duration and intensity, particularly for studies published from 1986 onwards [6]. Also, a systematic review of 16 studies conducted in various industries, with different study designs, did not identify a consistent association between having ever been exposed to formaldehyde by inhalation and mortality from lymphohematopoietic malignancies, including HL, MM, myeloid leukaemia, monocytic leukaemia, and lymphatic leukaemia [16]. This study observed that animal studies failed to detect detrimental effects on the lymphohematopoietic organs and a biologically plausible mechanism linking the absorption of formaldehyde, its systemic distribution, and in vivo genotoxicity [16].

Regarding the support from animal and mechanistic studies, despite some interpretative concerns, the IARC Monograph No. 100 F noted an excess of lymphohematopoietic malignancies among Wistar rats treated with formaldehyde in inhalation and oral studies [1]. In vivo and in vitro mechanistic evidence exists for its genotoxicity in human nasal tissues, while the evidence is moderate for bone marrow stem cells and circulating lymphocytes [1]. Indeed, the metabolism of formaldehyde to formic acid by alcohol dehydrogenase 5 (ADH5) and aldehyde dehydrogenase 2 (ALDH2) results in the formation of reactive oxygen species, which can cause inflammation and damage DNA [17]. Besides, the genetic ADH5 and ALDH2 deficiency in mice, a condition common among humans, leads to increased circulating levels of formaldehyde, which might then affect the bone marrow and cause cancer in the lymphatic tissue and solid organs [18]. Accordingly, the frequency of micronuclei and the level of formaldehyde-albumin adducts increased with cumulative exposure in peripheral blood lymphocytes of formaldehyde-exposed workers [19, 20]. Epigenetic changes have also been described through interference with DNA methylation, leading to hypermethylation of oncosuppressor genes, thereby favouring the neoplastic cell transformation [21].

Consistent with the U.S. NCI formaldehyde study [2, 5], we did not observe a linear trend in MM risk by cumulative exposure or average exposure intensity. However, we explored several other exposure metrics, and three, namely probability, frequency, and duration of exposure, had clear and significant upward trends. It is known from toxicological studies that chronic effects result from the interplay of dose, toxicokinetics, i.e. the half-life of a specific chemical in the organism, and time, i.e. the frequency of exposure. When exposure becomes close to continuous, even low doses might result in chronic toxicological effects [22]. As formaldehyde’s half-life in the organisms is typically short, the summation of effects from small, almost continuous doses might plausibly account for the observed inconsistent dose-response trend with intensity or cumulative exposure level.

Limitations of some published reports have been highlighted [16], including the lack of exposure assessment, which has frequently allowed exploration of generic exposure categorisations but not trends, and poor evaluation of confounders such as smoking and concurrent exposures. We tested the potential confounding of cigarette smoking and alcohol; however, neither was reported as a risk factor for multiple myeloma [23], and we did not find a difference in the prevalence of smokers or alcohol drinkers between cases and controls (Table 1). Therefore, we decided not to include them as covariates in the logistic regression models. Regarding potential occupational confounders, we explored farm work and solvents, both known risk factors for haematological malignancies, including multiple myeloma [24]. However, neither showed an association with risk of lymphoma or its subtypes in this study, nor did their inclusion reduce the residual variance of the regression models or change the risk estimates. IARC considers the evidence as limited for benzene as an aetiological agent for multiple myeloma [25]; however, we did not assess benzene exposure in this dataset and there was no association with MM and HL risk in a previous study [26]. Besides, in the NCI cohort study, risks associated with formaldehyde exposure did not change after controlling for benzene exposure [2].

The retrospective study design is a reason for concern when interpreting results from case-control studies, and it applies to the present study as well. The difference in participation rate between cases and controls is another limitation, common to most case-control studies. We do not have details on differences between participants and refusals for confounding factors or formaldehyde exposure. We relied on multivariate analysis to adjust for confounders, but we cannot rule out residual selection bias or confounding. Information bias was unlikely, as, at least in Italy, there was no widespread concern or media interest in formaldehyde during the data-gathering phase of this study. Self-reported information corroborated literature data [11–14], questionnaire information on exposure circumstances, and personal experience in assessing exposure, but was not the exclusive source of information. Therefore, we are confident that differential exposure reporting by case-control status was unlikely, and exposure misclassification might have been equally distributed between cases and controls, driving the potential bias towards the null. Still, our positive findings were based on small numbers, which is a further reason for concern. Finally, as we conducted multiple comparisons, some significant findings would be expected to occur by chance. Although partially consistent with some literature reports, we cannot rule out chance as an explanation for our findings.

Our paper also has several strengths. Based on statistical power calculations, our study size included a number of cases of the most prevalent B-cell lymphoma subtypes, which was enough to estimate Odds Ratios for ever exposure to formaldehyde ranging from 1.6 to 2.3. All cases were histologically confirmed. Also the multicenter study setting covered northern, central, and southern Italian regions, as well as a major island. Furthermore, the questionnaire included complete work histories and additional information on several exposures of a priori interest as well as major confounders.

To conclude, the results of our study suggest a possible role of daily exposure to formaldehyde, prolonged for 21 years or more, in the aetiology of multiple myeloma. Large occupational cohorts of workers with high formaldehyde exposure, and a follow-up prolonged enough to detect the number of incident lymphohaematopoietic malignancies required to conduct an accurate analysis, are not expected to become available in the future. Therefore, the best strategy to confirm or discard the hypothesis linking formaldehyde to an increased risk of ML and/or HL might be to access a large series of cases with complete work histories and the support of state-of-the-art exposure assessment.

Note: *cut-offs between intensity categories were estimated based on data from the literature (references No. 11–14) and questionnaire on work circumstances (indoor/outdoor, shop size, ventilation, use of personal protective equipment, direct/bystander exposure, and other were relevant information).

## Supplementary Information

Additional file 1. PCocco etal_Formaldehyde additional file 1.docx. Inclusion and exclusion criteria for hospital controls

Additional file 2. PCocco etal_Formaldehyde additional file 2.docx. Risk of lymphoma and subtypes by top frequency of exposure to formaldehyde

Additional file 3. PCocco etal_Formaldehyde additional file 3.docx. Risk of lymphoma and subtypes by average intensity of exposure to formaldehyde

Additional file 4. PCocco etal_Formaldehyde additional file 4.docx. Risk of lymphoma and subtypes by top intensity of exposure to formaldehyde

Additional file 5. PCocco etal_Formaldehyde additional file 5.docx. Risk of lymphoma and subtypes by top intensity of exposure to formaldehyde. Medium and high exposure intensity categories are combined

Additional file 6. PCocco etal_Formaldehyde additional file 6.docx. Risk of lymphoma and subtypes by years of exposure to formaldehyde

Additional file 7. PCocco etal_Formaldehyde additional file 7.docx. Risk of lymphoma and subtypes by cumulative exposure to formaldehyde (quartiles)

Additional file 8. PCocco etal_Formaldehyde additional file 8.docx. Risk of MM and HL and ever exposure to formaldehyde by latency

## Data Availability

Data are stored on the figshare repository and are publicly available (doi: 10.48420/29591138).

## References

[1] International Agency for Research on Cancer. IARC monographs on the evaluation of carcinogenic risks to humans. Chemical agents and related occupations. Volume 100F. Lyon, France: IARC; 2012. pp. 401–36. PMC4781612 23189753

[2] Beane Freeman LE, Blair A, Lubin JH, Stewart PA, Hayes RB, Hoover RN, Hauptmann M. Mortality from Lymphohematopoietic malignancies among workers in formaldehyde industries: the National cancer Institute cohort. J Natl Cancer Inst. 2009;101(10):751–61. 19436030 10.1093/jnci/djp096 PMC2684555

[3] Pottern LM, Heineman EF, Olsen JH, Raffn E, Blair A. Multiple myeloma among Danish women: employment history and workplace exposures. Cancer Causes Control. 1992;3(5):427–32. 1525323 10.1007/BF00051355

[4] Pira E, Romano C, Verga F, La Vecchia C. Mortality from Lymphohematopoietic neoplasms and other causes in a cohort of laminated plastic workers exposed to formaldehyde. Cancer Causes Control. 2014;25(10):1343–9. 25053406 10.1007/s10552-014-0440-0

[5] Checkoway H, Dell LD, Boffetta P, Gallagher AE, Crawford L, Lees PS, Mundt KA. Formaldehyde exposure and mortality risks from acute myeloid leukemia and other Lymphohematopoietic malignancies in the US National cancer Institute cohort study of workers in formaldehyde industries. J Occup Environ Med. 2015;57(7):785–94. 26147546 10.1097/JOM.0000000000000466 PMC4479664

[6] Catalani S, Donato F, Madeo E, Apostoli P, De Palma G, Pira E, Mundt KA, Boffetta P. Occupational exposure to formaldehyde and risk of non-Hodgkin lymphoma: a meta-analysis. BMC Cancer. 2019;19(1):1245. 31870335 10.1186/s12885-019-6445-z PMC6929467

[7] Protano C, Buomprosco G, Cammalleri V, Pocino RN, Marotta D, Simonazzi S, Cardoni F, Petyx M, Iavicoli S, Vitali M. The carcinogenic effects of formaldehyde occupational exposure: A systematic review. Cancers (Basel). 2021;14(1):165. 35008329 10.3390/cancers14010165 PMC8749969

[8] Morton LM, Slager SL, Cerhan JR, Wang SS, Vajdic CM, Skibola CF, Bracci PM, de Sanjosé S, Smedby KE, Chiu BC, et al. Etiologic heterogeneity among Non-Hodgkin lymphoma subtypes: the interlymph Non-Hodgkin lymphoma subtypes project. J Natl Cancer Inst Monogr. 2014;48:130–44. 10.1093/jncimonographs/lgu013 PMC4155467 25174034

[9] Campo E, Swerdlow SH, Harris NL, Pileri S, Stein H, Jaffe ES. The 2008 WHO classification of lymphoid neoplasms and beyond: evolving concepts and practical applications. Blood. 2011;117:5019–32. 21300984 10.1182/blood-2011-01-293050 PMC3109529

[10] Jaffe ES, Harris NL, Stein H, Vardiman JW. World health organization classification of tumours: pathology and genetics, tumours of hematopoietic and lymphoid tissues. Lyon, France: IARC; 2001.

[11] International Agency for Research on Cancer. IARC monographs on the evaluation of carcinogenic risks to humans. Wood dust and formaldehyde. Volume 62. Lyon, France: International Agency for Research on Cancer; 1995. pp. 217–364.

[12] Committee on Review of EPA’s 2022 Draft Formaldehyde Assessment, Board on Environmental Studies and Toxicology, Division on Earth and Life Studies. Review of EPA’s 2022 Draft Formaldehyde Assessment Washington, DC: National Academies, 2023. In: https://www.nap.edu/catalog/27153 (accessed 26 December 2023). 38359153

[13] Dubey SK, Das P, Formaldehyde. Risk assessment, environmental, and health hazard. Chapter 14. In: Singh J, Kaushik RD, Chawla M, eds. Hazardous Gases. London, UK: Academic Press, 2021 pp 169–182. In: https://linkinghub.elsevier.com/retrieve/pii/B9780323898577000268 (Accessed 13 May 2024).

[14] Cammalleri V, Pocino RN, Marotta D, Protano C, Sinibaldi F, Simonazzi S, Petyx M, Iavicoli S, Vitali M. Occupational scenarios and exposure assessment to formaldehyde: A systematic review. Indoor Air. 2022;32:e12949. 34708443 10.1111/ina.12949 PMC9298394

[15] Hayes RB, Blair A, Stewart PA, Herrick RF, Mahar H. Mortality of U.S. Embalmers and funeral directors. Am J Ind Med. 1990;18(6):641–52. 2264563 10.1002/ajim.4700180603

[16] Vincent MJ, Fitch S, Bylsma L, Thompson C, Rogers S, Britt J, Wikoff D. Assessment of associations between inhaled formaldehyde and Lymphohematopoietic cancer through the integration of epidemiological and toxicological evidence with biological plausibility. Toxicol Sci. 2024;199(2):172–93. 38547404 10.1093/toxsci/kfae039 PMC11131035

[17] Saito Y, Nishio K, Yoshida Y, Niki E. Cytotoxic effect of formaldehyde with free radicals via increment of cellular reactive oxygen species. Toxicology. 2005;210(2–3):235–45. 15840437 10.1016/j.tox.2005.02.006

[18] Dingler FA, Wang M, Mu A, Millington CL, Oberbeck N, Watcham S, Ponte LB, Kamimae-Lanning AN, Langevin F, Nadler C, et al. Two aldehyde clearance systems are essential to prevent lethal formaldehyde accumulation in mice and humans. Mol Cell. 2020;80(6):996–1012. 33147438 10.1016/j.molcel.2020.10.012 PMC7758861

[19] Yu LQ, Jiang SF, Leng SG, He FS, Zheng YX. [Early genetic effects on workers occupationally exposed to formaldehyde]. Zhonghua Yu Fang Yi Xue Za Zhi. 2005;39(6):392–5. [in Chinese]. 16329798

[20] Guo Y-J, Lin D-F, Yi J-H, Kuang D, Deng H-X, Li X-H, Zhang ZH, Wu TC. [The increase of micronuclei frequencies of peripheral blood lymphocyte in plywood workers exposed accumulatively to formaldehyde]. Zhonghua Lao Dong Wei Sheng Zhi Ye Bing Za Zhi 201;30(1):17–20. [in Chinese]. 22730682

[21] Chappell G, Pogribny IP, Guyton KZ, Rusyn I. Epigenetic alterations induced by genotoxic occupational and environmental human chemical carcinogens: A systematic literature review. Mutat Res Rev Mutat Res. 2016;768:27–45. 27234561 10.1016/j.mrrev.2016.03.004 PMC4884606

[22] Rozman KK, Dull J. Dose and time as variables of toxicity. Toxicology. 2000;40:169–78. 10.1016/s0300-483x(99)00204-8 10781885

[23] Sergentanis TN, Zagouri F, Tsilimidos G, Tsagianni A, Tseliou M, Dimopoulos MA, Psaltopoulou T. Risk Factors for Multiple Myeloma: A Systematic Review of Meta-Analyses. Clin Lymphoma Myeloma Leuk 2015;15(10):563-77.e1-3. 26294217 10.1016/j.clml.2015.06.003

[24] Perrotta C, Staines A, Cocco P. Multiple Myeloma and farming. A systematic review of 30 years of research. Where next? J Occup Med Toxicol 2008 17;3:27. 10.1186/1745-6673-3-27 PMC2628921 19014617

[25] International Agency for Research on Cancer. IARC monographs on the evaluation of carcinogenic risks to humans. Benzene. Volume 120. Lyon, France: IARC; 2018. pp. 37–297.

[26] Cocco P, t’Mannetje A, Fadda D, Melis M, Becker N, de Sanjosé S, Foretova L, Mareckova J, Staines A, Kleefeld S, et al. Occupational exposure to solvents and risk of lymphoma subtypes: results from the Epilymph case-control study. Occup Environ Med. 2010;67(5):341–7. 20447988 10.1136/oem.2009.046839

