# Supplementary material for: Occupational exposure to formaldehyde and risk of lymphoma subtypes: results of a multicentre Italian case-control study
Source: Environ Health. 2025 Oct 27;24:82. doi: 10.1186/s12940-025-01232-0 (PMC12557863; doi:10.1186/s12940-025-01232-0)
Supplement: Supplementary file 1 — Additional file 1. PCocco etal_Formaldehyde additional file 1.docx. Inclusion and exclusion criteria for hospital controls [file 12940_2025_1232_MOESM1_ESM.docx]

Additional file 1

Exclusion and inclusion criteria for hospital controls:

**a. Reasons for exclusion.** Patients admitted to hospital for one or more of the following diagnoses:

1. Malignant neoplasms (any).
2. AIDS.
3. Autoimmune diseases.
4. Allergy and atopy.
5. Viral hepatitis.
6. Transplanted patients.
7. Pre-neoplastic haematological diseases (MGUS [Monoclonal Gammopathy of Unknown Significance], bone marrow aplasia, myeloproliferative and myelodysplatic syndromes).

**b. Inclusion criteria:**

- 1. Non-neoplastic pazients (or with histologically benign neoplams, such as mammary fibroadenomas and prostatic adenomas) from general surgery departments (with indication of diagnosis and date of surgery and general anaesthesia).
  2. Trauma pazients.
  3. Pazients suffering from gastrointestinals disease (except Chron’s disease).
  4. Patients from ophtalmology departments.
  5. Pazients suffering from cardiovascular diseases.
  6. Pazients from haematology day clinics unaffected by neoplastic or pre-neoplastic diseases.
