# Supplementary material for: Occupational exposure to formaldehyde and risk of lymphoma subtypes: results of a multicentre Italian case-control study
Source: Environ Health. 2025 Oct 27;24:82. doi: 10.1186/s12940-025-01232-0 (PMC12557863; doi:10.1186/s12940-025-01232-0)
Supplement: Supplementary file 2 — Additional file 2. PCocco etal_Formaldehyde additional file 2.docx. Risk of lymphoma and subtypes by top frequency of exposure to formaldehyde [file 12940_2025_1232_MOESM2_ESM.docx]

**Additional file 2.** Risk of lymphoma and its most represented subtypes by top frequency of exposure to formaldehyde. Covariates in the logistic regression model include age, sex, study centre, and education.

| *Case subset* | *Unexposed* | *Top frequency* | | | |
| --- | --- | --- | --- | --- | --- |
|  |  | *1-3 times/month* | *1-4 times/week* | *daily* | *p* test for trend |
|  | *Cases/controls* | *Cases/Ctls OR 95%CI* | *Cases/Ctls OR 95% CI* | *Cases/Ctls OR 95% CI* |  |
| *All lymphomas* | 686/640 | 40/27 1.2 0.75 - 2.06 | 89/69 1.1 0.79 - 1.56 | 52/38 1.2 0.75 - 1.80 | 0.052 |
| *Non-Hodgkin’s lymphoma* | 391/640 | 18/27 1.0 0.54 - 1.87 | 43/69 1.0 0.66 - 1.51 | 28/38 1.2 0.70 - 1.97 | 0.149 |
| *B-cell lymphoma* | 378/640 | 20/27 1.1 0.57 - 1.94 | 43/69 1.0 0.63 - 1.47 | 26/38 1.1 0.62 - 1.82 | 0.310 |
| *Diffuse Large B-cell lymphoma* | 84/640 | 4/27 1.0 0.32 - 2.93 | 10/69 1.0 0.48 - 2.04 | 7/38 1.2 0.51 - 2.86 | 0.325 |
| *Follicular lymphoma* | 75/640 | 30/27 0.9 0.26 - 3.14 | 5/69 0.3 0.22 - 1.05 | 4/38 0.9 0.31 - 2.67 | 0.363 |
| *Chronic Lymphocytic Leukaemia* | 68/640 | 3/27 0.8 0.22 - 2.66 | 9/69 1.2 0.56 - 2.63 | 1/38 0.2 0.03 - 1.82 | 0.386 |
| *Multiple Myeloma* | 65/640 | 8/27 2.3 0.95 - 5.60 | 14/69 1.8 0.91 - 3.57 | 8/38 2.0 0.86 - 4.82 | 0.020 |
| *Hodgkin’s lymphoma* | 140/640 | 8/27 1.8 0.72 - 4.39 | 22/69 1.4 0.80 - 2.59 | 11/38 1.1 0.50 - 2.30 | 0.146 |
