# Supplementary material for: Occupational exposure to formaldehyde and risk of lymphoma subtypes: results of a multicentre Italian case-control study
Source: Environ Health. 2025 Oct 27;24:82. doi: 10.1186/s12940-025-01232-0 (PMC12557863; doi:10.1186/s12940-025-01232-0)
Supplement: Supplementary file 3 — Additional file 3. PCocco etal_Formaldehyde additional file 3.docx. Risk of lymphoma and subtypes by average intensity of exposure to formaldehyde [file 12940_2025_1232_MOESM3_ESM.docx]

**Additional file 3.** Risk of lymphoma and its most represented subtypes by average intensity of exposure to formaldehyde. Covariates in the logistic regression model include age, sex, study centre, and education.

.

| Case Subset | Unexposed | *Average intensity* | | | |
| --- | --- | --- | --- | --- | --- |
|  |  | *Low* | *Medium* | *High* | *p* test for trend |
|  | *Cases/controls* | *Cases/ctls OR 95%CI* | *Cases/ctls OR 95%CI* | *Cases/ctls OR 95%CI* |  |
| All lymphomas | 686/640 | 103/86 1.0 0.75-1.43 | 66/36 1.7 1.10-2.64 | 12/12 1.1 0.48-2.53 | 0.172 |
| Non-Hodgkin’s lymphoma | 391/640 | 56/86 1.0 0.71-1.53 | 29/36 1.3 0.76-2.23 | 4/12 0.7 0.21-2.15 | 0.878 |
| B-cell lymphoma | 378/640 | 61/86 1.2 0.78-1.71 | 24/36 1.1 0.60-1.89 | 4/12 0.7 0.21-2.27 | 0.739 |
| Diffuse Large B-cell lymphoma | 84/640 | 17/86 1.5 0.79-2.75 | 2/36 0.5 0.11-2.14 | 2/12 1.3 0.26-6.19 | 0.769 |
| Follicular lymphoma | 75/640 | 10/86 1.1 0.53-2.40 | 2/36 0.4 0.10-1.86 | 0/12 - - | 0.152 |
| Chronic Lymphocytic Leukaemia | 68/640 | 6/86 0.6 0.22-1.48 | 5/36 1.2 0.40-3.65 | 2/12 3.4 0.67-17.2 | 0.855 |
| Multiple Myeloma | 65/640 | 20/86 2.0 1.05-3.73 | 10/36 2.9 1.26-6.85 | 0/12 - - | 0.040 |
| Hodgkin’s lymphoma | 140/640 | 19/86 0.9 0.49-1.62 | 17/36 2.0 0.99-4.09 | 5/12 2.1 0.62-7.00 | 0.055 |
