# Supplementary material for: Occupational exposure to formaldehyde and risk of lymphoma subtypes: results of a multicentre Italian case-control study
Source: Environ Health. 2025 Oct 27;24:82. doi: 10.1186/s12940-025-01232-0 (PMC12557863; doi:10.1186/s12940-025-01232-0)
Supplement: Supplementary file 4 — Additional file 4. PCocco etal_Formaldehyde additional file 4.docx. Risk of lymphoma and subtypes by top intensity of exposure to formaldehyde [file 12940_2025_1232_MOESM4_ESM.docx]

**Additional file 4.** Risk of lymphoma and its most represented subtypes by top intensity of exposure to formaldehyde. Covariates in the logistic regression model include age, sex, study centre, and education.

.

| Case Subset | Unexposed | *Top intensity* | | | |
| --- | --- | --- | --- | --- | --- |
|  |  | *Low* | *Medium* | *High* | *p* test for trend |
|  | *Cases/controls* | *Cases/ctls OR 95%CI* | *Cases/ctls OR 95%CI* | *Cases/ctls OR 95%CI* |  |
| All lymphomas | 686/640 | 38/29 1.1 0.63-1.74 | 105/75 1.2 0.88-1.68 | 38/30 1.1 0.66-1.77 | 0.074 |
| Non-Hodgkin’s lymphoma | 391/640 | 20/29 1.0 0.54-1.80 | 49/75 1.1 0.73-1.62 | 20/30 1.0 0.56-1.83 | 0.133 |
| B-cell lymphoma | 378/640 | 23/29 1.1 0.59-1.90 | 50/75 1.1 0.72-1.61 | 16/30 0.8 0.43-1.53 | 0.337 |
| Diffuse Large B-cell lymphoma | 84/640 | 6/29 1.3 0.50-3.33 | 10/75 1.0 0.46-1.95 | 5/30 1.0 0.39-2.82 | 0.208 |
| Follicular lymphoma | 75/640 | 4/29 1.1 0.36-3.24 | 5/75 0.6 0.22-1.49 | 3/30 0.8 0.23-2.64 | 0.340 |
| Chronic Lymphocytic Leukaemia | 68/640 | 3/29 0.7 0.19-2.31 | 7/75 0.9 0.39-2.15 | 3/30 0.9 0.26-3.07 | 0.369 |
| Multiple Myeloma | 65/640 | 8/29 1.9 0.77-4.50 | 21/75 2.8 1.51-5.10 | 1/30 0.4 0.05-2.62 | 0.045 |
| Hodgkin’s lymphoma | 140/640 | 7/29 1.4 0.54-3.66 | 23/75 1.2 0.69-2.12 | 11/30 1.8 0.82-4.10 | 0.161 |
