# Supplementary material for: Occupational exposure to formaldehyde and risk of lymphoma subtypes: results of a multicentre Italian case-control study
Source: Environ Health. 2025 Oct 27;24:82. doi: 10.1186/s12940-025-01232-0 (PMC12557863; doi:10.1186/s12940-025-01232-0)
Supplement: Supplementary file 5 — Additional file 5. PCocco etal_Formaldehyde additional file 5.docx. Risk of lymphoma and subtypes by top intensity of exposure to formaldehyde. Medium and high exposure intensity categories are combined [file 12940_2025_1232_MOESM5_ESM.docx]

**Additional file 5.** Risk of lymphoma and its most represented subtypes by top intensity of exposure to formaldehyde. Covariates in the logistic regression model include age, sex, study centre, and education. Medium and high exposure intensity categories are combined.

| Case Subset | Unexposed | *Top intensity* | | |
| --- | --- | --- | --- | --- |
|  |  | *Low* | *Medium-high* | *p* test for trend |
|  | *Cases/controls* | *Cases/ctls OR 95%CI* | *Cases/ctls OR 95%CI* |  |
| All lymphomas | 686/640 | 38/29 1.0 0.59-1.66 | 143/105 1.3 0.96-1.72 | 0.908 |
| Non-Hodgkin’s lymphoma | 391/640 | 20/29 0.9 0.48-1.66 | 69/105 1.1 0.80-1.63 | 0.724 |
| B-cell lymphoma | 378/640 | 23/29 1.0 0.57-1.92 | 66/105 1.1 0.77-1.60 | 0.991 |
| Diffuse Large B-cell lymphoma | 84/640 | 6/29 1.2 0.47-3.30 | 15/105 1.2 0.64-2.25 | 0.974 |
| Follicular lymphoma | 75/640 | 4/29 1.0 0.33-3.28 | 8/105 0.7 0.32-1.60 | 0.280 |
| Chronic Lymphocytic Leukaemia | 68/640 | 3/29 0.7 0.19-2.64 | 10/105 1.0 0.43-2.12 | 0.706 |
| Multiple Myeloma | 65/640 | 8/29 1.7 0.68-4.31 | 22/105 2.2 1.23-4.12 | 0.010 |
| Hodgkin’s lymphoma | 140/640 | 7/29 1.4 0.54-3.83 | 34/105 1.5 0.91-2.49 | 0.184 |
