# Supplementary material for: Occupational exposure to formaldehyde and risk of lymphoma subtypes: results of a multicentre Italian case-control study
Source: Environ Health. 2025 Oct 27;24:82. doi: 10.1186/s12940-025-01232-0 (PMC12557863; doi:10.1186/s12940-025-01232-0)
Supplement: Supplementary file 6 — Additional file 6. PCocco etal_Formaldehyde additional file 6.docx. Risk of lymphoma and subtypes by years of exposure to formaldehyde [file 12940_2025_1232_MOESM6_ESM.docx]

**Additional file 6.** Risk of lymphoma and its most represented subtypes by quartiles of years of exposure to formaldehyde. Covariates in the logistic regression model include age, sex, study centre, and education.

| Case Subset | Unexposed | *Years of exposure* | | | | |
| --- | --- | --- | --- | --- | --- | --- |
|  |  | *Ist quartile* | *2nd quartile* | *3rd quartile* | *4th quartile* | *p* test for trend |
|  | *Cases/controls* | *Cases/ctls OR 95%CI* | *Cases/ctls OR 95%CI* | *Cases/ctls OR 95%CI* | *Cases/ctls OR 95%CI* |  |
| All lymphomas | 686/640 | 52/37 1.2 0.79-1.92 | 41/32 1.1 0.66-1.74 | 38/36 0.9 0.55-1.43 | 50/29 1.5 0.90-2.35 | 0.079 |
| Non-Hodgkin’s lymphoma | 391/640 | 24/37 1.2 0.68-2.03 | 19/32 1.0 0.55-1.84 | 19/36 0.8 0.44-1.40 | 27/29 1.3 0.73-2.18 | 0.171 |
| B-cell lymphoma | 378/640 | 23/37 1.2 0.69-2.14 | 19/32 1.0 0.56-1.91 | 20/36 0.7 0.40-1.29 | 27/29 1.1 0.65-1.98 | 0.357 |
| Diffuse Large B-cell lymphoma | 84/640 | 7/37 1.4 0.31-3.31 | 7/32 1.5 0.62-3.69 | 5/36 0.9 0.34-2.40 | 2/29 0.4 0.10-1.84 | 0.372 |
| Follicular lymphoma | 75/640 | 5/37 1.3 0.49-3.54 | 1/32 0.3 0.03-2.03 | 2/36 0.4 0.10-1.86 | 4/29 0.9 0.31-2.75 | 0.359 |
| Chronic Lymphocytic Leukaemia | 68/640 | 1/37 0.3 0.05-2.60 | 6/32 1.9 0.72-5.02 | 4/36 0.9 0.29-2.57 | 2/29 0.4 0.10-1.89 | 0.271 |
| Multiple Myeloma | 65/640 | 5/37 1.9 0.69-5.46 | 5/32 1.6 0.55-4.69 | 6/36 1.2 0.44-3.01 | 14/29 3.1 1.51-6.26 | 0.005 |
| Hodgkin’s lymphoma | 140/640 | 20/37 1.4 0.75-2.70 | 10/32 1.1 0.51-2.51 | 6/36 1.1 0.44-2.85 | 5/29 2.6 0.92-7.29 | 0.138 |
