# Supplementary material for: Occupational exposure to formaldehyde and risk of lymphoma subtypes: results of a multicentre Italian case-control study
Source: Environ Health. 2025 Oct 27;24:82. doi: 10.1186/s12940-025-01232-0 (PMC12557863; doi:10.1186/s12940-025-01232-0)
Supplement: Supplementary file 7 — Additional file 7. PCocco etal_Formaldehyde additional file 7.docx. Risk of lymphoma and subtypes by cumulative exposure to formaldehyde (quartiles) [file 12940_2025_1232_MOESM7_ESM.docx]

**Additional file 7.** Risk of lymphoma and its most prevalent subtypes by quartiles of cumulative formaldehyde exposure. Covariates in the logistic regression model include age, sex, study centre, and education.

| Case Subset | Unexposed | *Cumulative exposure* | | | | |
| --- | --- | --- | --- | --- | --- | --- |
|  |  | *1st quartile* | *2^nd^ quartile* | *3rd quartile* | *4th quartile* | *p* test for trend |
|  | *Cases/controls* | *Cases/ctls OR 95%CI* | *Cases/ctls OR 95%CI* | *Cases/ctls OR 95%CI* | *Cases/ctls OR 95%CI* |  |
| All lymphomas | 686/640 | 48/31 1.3 0.82-2.11 | 45/33 1.2 0.73-1.87 | 41/38 0.9 0.31-1.48 | 47/32 1.2 0.77-1.96 | 0.107 |
| Non-Hodgkin’s lymphoma | 391/640 | 25/31 1.4 0.83-2.53 | 17/33 0.8 0.43-1.45 | 27/38 1.2 0.69-1.98 | 20/32 0.9 0.48-1.54 | 0.253 |
| B-cell lymphoma | 378/640 | 24/31 1.4 0.80-2.56 | 25/33 1.1 0.63-1.92 | 19/38 0.8 0.44-1.45 | 21/32 0.8 0.46-1.48 | 0.397 |
| Diffuse Large B-cell lymphoma | 84/640 | 8/31 1.9 0.80-4.34 | 3/33 0.6 0.17-1.90 | 7/38 1.4 0.57-3.22 | 3/32 0.3 0.17-1.98 | 0.315 |
| Follicular lymphoma | 75/640 | 6/31 1.9 0.73-4.77 | 4/33 1.0 0.34-2.91 | 0/38 - - | 2/32 0.4 0.10-1.84 | 0.161 |
| Chronic Lymphocytic Leukaemia | 68/640 | 5/31 1.6 0.57-4.48 | 1/33 0.3 0.03-1.88 | 2/38 0.5 0.11-2.19 | 5/32 1.2 0.42-3.13 | 0.298 |
| Multiple Myeloma | 65/640 | 3/31 1.1 0.29-3.84 | 13/33 3.6 1.69-7.64 | 7/38 1.6 0.66-4.09 | 7/32 1.5 0.63-3.77 | 0.013 |
| Hodgkin’s lymphoma | 140/640 | 13/31 1.1 0.51-2.35 | 14/33 2.0 0.97-4.14 | 4/38 0.5 0.16-1.43 | 10/32 2.7 1.19-6.20 | 0.130 |
