# Supplementary material for: Occupational exposure to formaldehyde and risk of lymphoma subtypes: results of a multicentre Italian case-control study
Source: Environ Health. 2025 Oct 27;24:82. doi: 10.1186/s12940-025-01232-0 (PMC12557863; doi:10.1186/s12940-025-01232-0)
Supplement: Supplementary file 8 — Additional file 8. PCocco etal_Formaldehyde additional file 8.docx. Risk of MM and HL and ever exposure to formaldehyde by latency [file 12940_2025_1232_MOESM8_ESM.docx]

**Additional file 8**. MM and HL risk and ever exposure to formaldehyde by years since exposure ceased.

|  | *Multiple myeloma* | | *Hodgkin’s lymphoma* | |
| --- | --- | --- | --- | --- |
|  | *Unexposed* | *Ever Exposed* | *Unexposed* | *Ever Exposed* |
|  | *Cases/ctrls* | *Cases/ctrls OR 95% CI* | *Cases/ctrls* | *Cases/ctrls OR 95% CI* |
| *≤ 9 years* | 65/640 | 3/16 1.9 0.50-7.02 | 140/640 | 4/16 0.8 0.22-2.60 |
| *10-14 years* | 65/640 | 3/11 2.0 0.71-5.88 | 140/640 | 7/11 4.2 1.35-13.2 |
| *15-19 years* | 65/640 | 4/13 1.9 0.54-6.40 | 140/640 | 0/13 - - |
| *≥ 20 years* | 65/640 | 11/35 1.7 0.78-3.82 | 140/640 | 4/35 1.0 0.31-3.01 |
